# Cannabis Withdrawal Induced Mania. A two year observational study of Hospital admissions from 2015 to 2019

**DOI:** 10.1192/j.eurpsy.2025.1067

**Published:** 2025-08-26

**Authors:** I. Ochandiano Recio, I. Pacchiarotti, A. Gimenez Palomo, H. Andreu Gracia, S. Salmerón Morales, J. I. Mena, E. Cesari, T. Fernandez-Plaza, L. Olivier, O. De Juan Viladegut

**Affiliations:** 1 Instituto de Neurociencias, Servicio de Psiquiatría y Salud Mental, Hospital Clinic de Barcelona, Barcelona, Spain

## Abstract

**Introduction:**

Bipolar disorder (BD) is a recurrent psychiatric illness characterized by manic, hypo-manic, depressive and mixed episodes. The endocannabinoid system (ECS) is a vast network of chemical signals and cellular receptors that are densely packed throughout the brain. It is involved in most critical central nervous system functions, such as learning and memory, but also mood regulation. Despite this, there is only anecdotal evidence on the potencial role of the ECS in the pathophysiology of BD.

**Objectives:**

This study aims to retrospectively assess clinical and sociodemographic variables of patients whose manic episode is temporally associated with the suspension of cannabis use, compared with the rest of a sample of manic patients admitted to an acude psychiatry ward. The objective of the study is to investigate the presence of a specific group of BD patients, with potential clinical and therapeutic implications.

**Methods:**

We retrospectively analysed all admitted patients to the acute psychiatry unit at Hospital Clinic of Barcelona from 2015 to 2019 and hospitalized for a manic episode. Patients with core manic symptoms started after cannabis withdrawal were included in the study. Cannabis Withdrawal Induced Mania (CWIM) group (n=20) was compared with the rest of bipolar manic patients in our sample according to several clinical and sociodemographic variables. Univariate and multivariate logistic regression models were performed.

**Results:**

between 2015 and 2019, 315 patients were admitted to the acute psychiatry unit with diagnosis of manic episode. In 282 of the cases, the history of cannabis use was documented. 20 of them meet criteria for CWIM. CWIM group patients were significantly more frequently men (p=0.015) and younger (p<0.001), were not married or in a relationship (p = 0.018) and less frequently had somatic illnesses (p=0.41). Patients with CWIM debuted with a first manic episode and had their first psychiatry admission at a significantly younger age (p=0.008 and p=0.004, respectively). Previous treatment with antipsychotic medication was significantly less frequent in the CWIM group (p=0.022). According to follow-up, there were no significant differences in relapse after three years (p=0.936).

**Conclusions:**

This study found a new clinical profile of patients, who are more suggestive to have a manic episode in context of cannabis withdrawal.

**Disclosure of Interest:**

None Declared

